# New York Academy of Medicine

**Published:** 1890-12

**Authors:** 


### NEW YORK ACADEMY OF MEDICINE.—SECTION ON ORTHOPEDIC SURGERY.

Stated meeting, October 17, 1890.

V. P. Gibney, M. D., in the chair.

### NON-UNION OF FRACTURED RADIUS.

Dr. C. A. Powers exhibited a patient in whom this condition had existed for many years, and also showed an extension apparatus which had given relief. The first fracture occurred twenty-nine years ago, at the junction of the middle and lower thirds. A refracture took place eighteen years later, and united with deformity and disability. The radical nerve had become involved in the callus, and this gave rise to such intense pain that she underwent an operation for its relief five years later, in which the bone was again fractured. All attempts to cause this fracture to unite failed. When she came under the care of the speaker in May of the present year, it was found that the carpus had slipped upwards with the lower fragment of the radius, and had caused the ulna to project very forcibly against the soft parts, giving rise to much pain in the region supplied by the ulnar nerve. As further operative measures were not deemed advisable, a simple extension apparatus was applied, and had answered admirably.

Dr. A. M. Phelps said that he thought it had been wisely decided not to subject the patient to further operation, as fractures of the radius and of the lower third of the tibia were peculiarly prone to non-union. Out of about 300 osteotomies, he had had only one case of non-union, and that was after an operation for the correction of an anterior tibial curve. Operations by himself and others had failed to bring about union. Thomas, of Liverpool, claimed that such fractures could be made to unite by pounding the parts with a mallet ; but in his experience this method had not proved successful, and he thought that where there was muscle between the ends of the bone, and the peculiar ivory-like condition of the ends of the bone, which was not uncommonly present, none of the methods heretofore proposed were likely to prove successful. He had very recently proposed and performed a new operation, which he thought might prove successful. It consisted in cutting down upon the ununited fracture, freshening the ends of the bone, and grafting in between them a part of the forearm of a dog, both patient and dog being secured in plaster of paris. When the graft had united firmly, the dog's leg would be amputated, and the skin flaps of the dog united to those of the patient.

### HIP-JOINT DISEASE AFTER TYPHOID FEVER.

Dr. J. Mc. G. Woodbury presented a girl of eleven years, who six months after a severe attack of typhoid fever was found to have some limitation of motion and pain at the right hip, with distension of the capsule. Flexion caused lordosis, and some pain. She was treated by counter-irritation over the joint, and a plaster of paris spica bandage, and was allowed to walk around upon a high patten, with crutches. Now, after a period of eight months, there was no pain.

### A CASE OF OSTEO-MALACIA.

Dr. Woodbury also presented a case of this nature. The patient had lived in Switzerland until twenty-six years of age, and had suffered considerably from exposure during the late war. On October 26, 1886, when forty-three years of age, he sustained a fracture of the surgical neck of the left humerus, and between that date and May 26, 1890, he received five other fractures, viz., two of the left humerus, two of the right humerus, and one of the left clavicle. Most of these fractures were caused by very slight falls. During the last three months, but more particularly since the first of last August, a tumor has been rapidly growing between the sides of the two fractures of the shaft of the right humerus. Two small tumors may be observed upon the clavicle, one at the point of the fracture, and the other to the inside of it. A specimen removed from the large tumor with a harpoon was sent to Dr. J. S. Ely for microscopical examination, and he reported that it contained "polyhedral cells, and occasional large spindle and giant cells." He adds, that this "speaks very strongly for sarcoma." A loud murmur, similar to that heard in aortic aneurism, is audible over the large tumor. Dr. Woodbury said that as in cases of tumor of the middle of the spinal cord, osteo-malacia, due to trophic disturbances, is one of the early symptoms concurrent with disturbances of sensation, he had referred the case to Dr. E. A. Starr, with the hope of learning more about the etiology of this interesting condition. Dr. Starr examined the patient on two or three occasions, the last time only a few days ago, and had reported that there was no central lesion of the cord. The patient had had no pain with the fractures, or upon re-setting these bones, and this, together with the fact that there had been no fractures of the lower extremity, seemed to favor the view that the condition was due to a syringe-myelia, or tumors of the cord.

Dr. Powers said that Dr. Woodbury's case of multiple fracture with tumors was very similar to a case of multiple sarcomata which he had recently presented to the Surgical Section.

Dr. V. P. Gibney thought the pulsation in the tumor might be due to the condition of the tumor itself; in other words, it might be a pulsating sarcoma.

### ANKLE JOINT DISEASE.

Dr. A. B. Judson presented a case of this disease which he said was interesting, because the child had suffered from this condition almost all her life. The disease began at the age of one year, and she is now about seven years old. Notwithstanding that she had been under mechanical treatment only two years, she had recovered with but little disability and deformity. There was considerable lateral motion at the ankle joint; extension was almost normal; flexion was arrested at about ninety degrees. Scars on both sides of the ankle showed where abscesses had opened spontaneously. There was a difference of one inch between the two calves, and the shortening amounted to only a small fraction of an inch. This result had been obtained by the use of a simple brace, and without resorting to any operation.

Dr. John Ridlon presented an astragalus, which had been removed by Dr. B. Farquhar Curtis from a child which had been brought to the speaker when only six weeks old. He had faithfully tried stretching, and the various retentive appliances, during a period of one and a half years. Dr. G. S. Huntington had then operated by Dr. A. M. Phelps' open method, but without improving the condition. The specimen which he presented was interesting on account of two bony prominences which it showed, and which apparently had been the obstacle to flexion of the foot.

### THE TREATMENT OF ANKLE-JOINT AND TARSAL DISEASE.

The paper of the evening, with the above title, was read by Dr. T. Halsted Myers, who also presented a patient illustrative of this subject.

Dr. Myers said that tubercular inflammation might attack, first, the synovial membrane, later the cartilage, and lastly the bone ; or the primary local focus might be in the bone.

While it was still confined to the synovial membrane, a number of surgeons recommended erasion. If it had attacked the bone, many more urged operative methods, irrespective of the general health of the patient. The author considered only the latter condition.

Simple incision was of no advantage, for we had no element of tension, as in acute processes, and we only opened new channels of infection, leaving the original disease unchanged.

The usual method of treatment, curetting the abscess walls and the sinuses, could not be expected to remove all disease, and would greatly increase the risk of absorption. The success which had been secured in some of these cases seemed to be due to the power of the antiseptic agent to render inert the bacilli which remained.

The rational method was to remove all the disease at once ; but apparently healthy bones contained tuberculous foci, and hence, it was a most difficult problem to know when to stop, and, in fact, this could not be determined at the time of operation. If all the disease were successfully removed, the duration of treatment was less than under conservative methods. The ultimate results were, however, less satisfactory. He had seen a considerable number of misshapen and atrophied feet after operative treatment, which were weak and painful, and required support to render them able to bear the weight of the body. He had not observed such results from conservative treatment. It was confessedly difficult to ascertain the ultimate results ; and although Dr. Shaffer had kindly placed the records of the New York Orthopedic Dispensary at his service, he had not been able in the short time at his disposal to do more in most of the cases than quote the histories.

The number of cases treated before July, 1888, was fifty-five, and of these he knew personally that at least twenty-one were cured. Five were cases of synovitis, and sixteen of osteitis. The average duration of treatment in the latter was twenty-one and a half months, the longest case being under treatment fifty-five months. The results in all were extremely good; yet, under careful private treatment, still better results should be expected.

From our knowledge of the various ways in which the bacilli of tuberculosis may be spread in the body, it would seem that a primary tubercular process in a joint must be extremely rare. Drs. Prudden, Northrup, Biggs, and Thacher, to whom he had written for information on this subject, all considered that these affections were generally secondary, but agreed that primary joint lesions did occur. The practical importance of this was that the danger of general infection from a joint lesion which was not interfered with surgically, was an entirely unknown and probably extremely small quantity.

Of the whole number treated (fifty-five), but three had died — one of diphtheria, one while tarsal disease was active, and the other, six months after, a note of "nearly cured" had been recorded. In neither of the latter was the cause of death stated. However, in Dr. Scudder's report of eighteen cases of excision, six deaths occurred; three were due to the operation, or its direct effects; another might have been; and the other two were from tuberculosis, but occurred one and two years after the operations.

The treatment of synovitis consisted in absolute protection of the joint from traumatism. In children, he considered a perineal crutch absolutely necessary while walking. Ordinary crutches were invariably laid aside at times, and the joint left unprotected. In adddition to this crutch, the foot should be protected by a splint, to avoid local injuries and to maintain a good position. There being no involuntary muscular spasm, while the disease was confined to the synovial membrane, traction was not necessary.

In cases of osteitis, the same protection of the joint was imperative, and if there were pain and spasm, indicating the necessity for traction, this could be applied at the ankle by means of a Dow's brace, or the apparatus of Dr. Sayre or Dr. Foster.

The application of adhesive plaster to a painful ankle required more care than a dispensary case was willing to give, especially when abscess was present. For this reason he had found it most serviceable to employ a leg brace, or plaster splint, worn constantly, and a perineal crutch for walking, which could be laid aside at night; or the Dow's brace, as modified by Dr. Shaffer, might be used.

Abscesses should be left entirely alone, and the sinuses simply kept aseptic. After the joint was considered cured, it was well to wear an ankle brace for some months, to prevent twists. The malpositions found in the acute stages were almost entirely due to muscular spasm, and did not require tenotomy, or other operative treatment.

In the later stages there might be bony changes, and these, if not painful or progressive, did not require treatment. However, if these conditions did exist, and yet there was no evidence of active disease, an attempt should be made to restore and preserve the normal relations of the parts.

The value of hygienic surrounding during the treatment of these cases could not be overestimated. His observations had been made on children only, and for contrast an extended series of cases in the adult would be very valuable. Without exception, every one of his cases of ankle-joint or tarsal osteitis in children had done well under conservative treatment, and he had yet to see the case which he would condemn to erasion or excision.

Dr. N. M. Shaffer said that his own experience led him to think that one point in Dr. Myers' paper should be particularly emphasized, *i. e.*, the necessity of absolute protection of the articulation. He had accomplished this in practice, whenever possible, by the use of a modification of Dow's brace, and had found that adhesive plaster was rarely required, as a well-fitting shoe made efficient counter-traction. He thought that the further removed the tuberculous joint was from the center of the body, the more benign was the disease, and the less danger of general infection; and he was inclined to speak more strongly of the conservative treatment of ankle-joint disease than of any other articulation in the body.

Dr. Ridlon thought these cases did well with the Dow's instrument; but with this, as with some others, we could not secure immobilization, but only protect the joint from the jar of walking. He had seen such excellent results in cases of suppurative ankle-joint disease, without any treatment whatever, that he often doubted how much of a good result could be attributed to the treatment received.

Dr. H. W. Berg said that he had had such good results in the treatment of phthisis by the administration of the bichloride of mercury in doses of one-twenty-fourth of a grain, three times a day, that he was inclined to believe the old theory, that tuberculosis was really a change in the syphilitic virus due to passing through several generations. He considered that splints like Dr. Judson's were imperfect, for, by taking their bearing from the outside of the foot, intra-articular pressure was increased. To diminish this pressure, the foot must be adducted and rotated inwards.

Dr. Phelps was of the opinion that the vast majority of these cases were cured by immobilization and relief of intra-articular pressure, but in suppurative cases he believed that the soundest and most scientific surgery demanded operative measures. If we could protect the hip-joint as well as the ankle-joint, we ought to get equally good results in hip diseases. He believed that these cases were inoculations of pathogenic germs on a diseased surface, and that they were purely local.

Dr. R. H. Sayre exhibited a splint which his father had devised for an adult with ankle-joint disease. He agreed with Dr. Ridlon, that it was difficult to apply traction at this joint, but he thought this splint solved the problem. His views regarding the prognosis and treatment of this disease were in accordance with those just expressed by Dr. Phelps.

Dr. Samuel Lloyd said that fifteen cases of adult ankle-joint disease had been treated in the New York Post-Graduate School by this so-called conservative method, but the relapses had been very frequent, and he thought this method was less likely to yield good results in adults than in children. In answer to questions from the chairman, he said that several of the cases were due to injury, and a number of them were suppurative, while four were recorded as synovitis. Two of the cases had been discharged as cured before 1883, and were known to be well in 1889.

Dr. Judson protested against the statement that cases of disease in the ankle should do equally well without treatment, although neglected cases of ankle-joint disease would have nothing like so bad a deformity as those at the hip.

Dr. H. L. Taylor also spoke about the different mechanical conditions present at the various joints. The weight of the limb exerted great leverage upon the joint, especially in a spasmodic condition of the muscles. It is more marked at the hip than at the knee, and very much more noticeable than at the ankle. He referred to a case of ankle-joint disease occurring in a distinctly phthisical subject, where the sinuses were treated by injections of a saturated solution of iodoform in ether. The beneficial effect upon the healing process was almost magical.

Dr. Gibney said that about ten years ago, the surgical section of the "Therapeutic Society" of this city spent about two years collecting data relative to the comparative results obtained by the operative and non-operative treatment of this condition; and the conclusion was, that the conservative method yielded the greatest number of useful ankles, even in cases where the foot was seen with cicatrices. There were two or three operative cases, having a high degree of equinus, and a stiffened and shortened joint, and one or two flail joints were also shown. In his experience, cases of adult ankle-joint disease relapsed again and again on the slightest provocation; later on, abscesses would appear; still later, pulmonary signs would develop, and then amputation would follow. As regards the mercurial treatment of tuberculous disease of the joint, he need only call attention to the fact that many years ago the routine treatment for these cases at the Hospital for Ruptured and Crippled, was one-twenty-fourth of a grain of the bichloride of mercury in tincture of bark, three times a day; and the results attained by this treatment were certainly far from striking.

